# Auditory DUM neurons in a bush-cricket: inhibited inhibitors

**DOI:** 10.1007/s00359-020-01438-2

**Published:** 2020-07-12

**Authors:** Andreas Stumpner, Silvia Gubert, Debbra Y. Knorr, Martin C. Göpfert

**Affiliations:** Department of Cellular Neurobiology, Johann-Friedrich-Blumenbach-Institute of Zoology and Anthropology, Georg-August-University of Göttingen, Julia-Lermontowa-Weg 3, 37077 Göttingen, Germany

**Keywords:** Insect, Hearing, Inhibition, Segmental interneurone, Immunohistochemistry

## Abstract

Thoracic ganglia of many hearing insects house the first level of auditory processing. In bush-crickets, the largest population of local auditory neurons in the prothoracic processing centre are dorsal unpaired median (DUM) neurons. It has been suggested that DUM neurons are inhibitory using γ-aminobutyric acid (GABA) as transmitter. Immunohistochemistry reveals a population of about 35–50 GABA-positive somata in the posterior medial cluster of the prothoracic ganglion. Only very few small somata in this cluster remain unstained. At least 10 neurites from 10 neurons can be identified. Intracellularly stained auditory DUM neurons have their soma in the cluster of median GABA positive cells and most of them exhibit GABA-immunoreactivity. Responses of certain DUM neurons show obvious signs of inhibition. Application of picrotoxin (PTX), a chloride-channel blocker in insects, changes the responses of many DUM neurons. They become broader in frequency tuning and broader or narrower in temporal pattern tuning. Furthermore, inhibitory postsynaptic potentials (IPSPs) may be replaced by excitatory postsynaptic potentials. Loss of an IPSP in the rising graded potential after PTX-application leads to a significant reduction of first-spike latency. Therefore, auditory DUM neurons receive effective inhibition and are the best candidates for inhibition in DUM neurons and other auditory interneurons.

**Electronic supplementary material:**

The online version of this article (10.1007/s00359-020-01438-2) contains supplementary material, which is available to authorized users.

## Introduction

Auditory systems retrieve information about carrier frequency, temporal pattern and direction of sounds (Hedwig and Pollack 2008). The respective information is extracted by auditory neurons. Fundamental questions in hearing research are the formation of specific properties of auditory neurons and the relevance of processed information for behavioural decisions. Insects are well suited to address these questions, since the populations of neurons relevant for certain tasks are smaller than in vertebrates and neurons often can be individually identified and characterized (e.g., Comer and Robertson 2001; Hedwig and Pollack 2008).

Auditory sensory neurons of most hearing insects are frequency selective and typically provide a rough copy of the temporal pattern, though modified by adaptation time constants (Gollisch and Herz 2004; Mason and Faure 2004). Projections of sensory cells in the auditory neuropils synapse onto local or intersegmental neurons, the latter forwarding the information directly to the brain, where recognition and directional decisions mainly take place (Nolen and Hoy 1984; Ronacher et al. 1986; Römer et al. 1988; Hedwig 2016). Before information is forwarded to the brain, however, local neurons on the first level of auditory processing assist in shaping the ascending information (Römer et al. 1981; Pollack 1988; Stumpner 2002). The most prominent example is the local omega neuron 1 from crickets (Casaday and Hoy 1977) and bush-crickets (Zhantiev and Korsunovskaya 1983). Due to the prominent reciprocal inhibition between the pair of omega-neurons (Selverston et al. 1985; Römer and Krusch 2000) it is mainly associated with directional processing (see, however, Wiese and Eilts-Grimm 1985; Römer and Krusch 2000; Reeve and Webb 2003; Zhang and Hedwig 2019 for potential other functions). Inhibition related not only to direction but also to frequency and pattern recognition exists in many auditory neurons of Orthoptera (Römer et al. 1981; Römer and Seikowski 1985; Stumpner et al. 1995; Stumpner 1998, 2002), but very few sources have been identified (Marquart 1985). One candidate class of inhibitory local neurons are DUM neurons (dorsal unpaired median; Hoyle et al. 1974) with small somata, most of which have GABA as transmitter in locusts (Watson 1986) and some other insects, while those with large somata have octopamine as transmitter (Bräuning and Pflüger 2001). Local auditory DUM neurons with small soma are described for grasshoppers (Stumpner and Ronacher 1991; Thompson and Siegler 1991) and bush-crickets (Lefebvre et al. 2018). In the bush-cricket *A. nigrovittata* a population of at least 15 DUM neurons provides a filter bank for carrier frequency processing (Lefebvre et al. 2018) and certain temporal parameters (Stumpner et al. 2019). They have been hypothesized to inhibit local or ascending auditory neurons in the prothoracic ganglion (Lefebvre et al. 2018), but it has not even been shown that they contain GABA as transmitter so far.

The bush-cricket *A. nigrovittata* with males and females singing at different carrier frequencies and with different temporal patterns has become a model case for auditory processing. The system has a—for bush-crickets—unusually large number of elements with auditory input in the prothorax (Stumpner and Molina 2006, Stumpner et al. 2019). Inhibition obviously plays a prominent role in shaping specific responses (Stumpner 1998, Molina and Stumpner 2005). To further our understanding of processing in this species we asked the following questions: Do all auditory DUM neurons of *A. nigrovittata* contain GABA as transmitter and therefore meet the expectation to act as inhibitory elements in the prothoracic network? If so, does immunostaining allow an estimation of the number of local GABAergic DUM neurons, which remained equivocal from a huge dataset with morphological and physiological characterization of DUM neurons? In addition, we used the mesothoracic ganglion as a control (no direct auditory input). Do DUM neurons that show signs of inhibition change their responses to auditory stimuli when inhibition is blocked thereby indicating that prominent inhibiting elements receive inhibition themselves?

## Material and methods

### Animals

Both sexes of the bush-cricket *A. nigrovittata* (Brunner von Wattenwyl) were studied and were mostly laboratory-reared from eggs collected from the laboratory culture each summer. Few animals were wild-captured from Northern Greece. From 74 nervous systems (mostly pro- and mesothoracic ganglia) used for immunohistochemistry, 11 were from females. Sixty eight animals were used for pharmacological experiments yielding 80 evaluated cells (56 from males, 24 from females).

### Recording and staining techniques

Recording and staining have been described in detail by Lefebvre et al. (2018) and Stumpner et al. (2019). Briefly, the animal was anaesthetized for about 3 min with CO_2_ and fixed ventral-side-up to a plastic holder with a wax–resin mixture. The legs were immobilized in an inverse standing position. The prothoracic ganglion was exposed and stabilized using a Ni–Cr spoon or a steel ring from below and a steel ring from above. A grain of dry collagenase (Sigma Aldrich, Darmstadt, Germany) was placed posteriorly on the air-exposed ganglion for 25 s, before adding saline for 10 min, followed by three washings with saline (Fielden 1960). The collagenase served to facilitate penetration of the ganglionic sheath by the glass capillary and the diffusion of picrotoxin into the neuropile. Thick-walled borosilicate glass capillaries were either filled with lucifer yellow CH (5% w/v in 0.5 M LiCl, Sigma Aldrich or Molecular Probes), with Alexa Hydrazide 488, 555 (both 10 mM in 200 mM KCl) or Alexa 633 (5 mM in 100 mM KCl; all Alexa dyes from Life Technologies, Darmstadt, Germany) or with CF633 (5 mM in 100 mM KCl; Biotium, Hayward, CA, USA). In some of the experiments combining intracellular staining and immunohistochemistry the capillary was filled with neurobiotin (Vector, Burlingame, USA). Recordings were amplified with a direct-current amplifier (NPI BA-1S, NPI, Tamm, Germany), and stored on a computer, with the program Spike2 using a sampling rate of 20 kHz/channel (CED power 1401, CED, Cambridge, UK). The dye was ionophoretically injected for 0.5–15 min with 0.5–2 nA hyperpolarizing current after physiological characterization of a neuron. Neurobiotin was injected with depolarizing current up to 1.6 nA. In case of picrotoxin application, ionophoresis was applied for up to 1 min with up to 1 nA during the diffusion time. After an experiment, the ganglia were excised and fixed in *para*-formaldehyde (4% in buffer, pH 7.4) for 1 h. If no immunostainings followed, the ganglia were dehydrated (15 min in 70, 90, 96 and 2 times 100% ethanol) after washing in PBS and cleared in methylsalicylate. Stained neurons were visualized with a confocal microscope (Leica SP2 AOBS or Leica SP8 AOBS, Wetzlar, Germany) and projections of confocal scans were calculated.

### Immunostaining

Immunostainings were performed with horizontal sections of freshly prepared ganglia. Several combinations of fixation time, washing in buffers with detergent and treating with enzymes were tested. In the finally successful variant, ganglia were fixed for 1 h at 4 °C in 4% PFA in 0.1 M phosphate buffer (PB) at pH 7.4. They then were washed five times for 15 min in phosphate buffered saline (PBS) at room temperature (RT) on a shaker. Then ganglia were embedded in 5% agarose (Sigma Aldrich), horizontally sectioned to between 60 µm and 280 µm and collected in wells (1 per specimen) in PBS with 1% Triton (PBST; Sigma Aldrich), where they remained for 36–60 h on a shaker at 4 °C. Then, for 3 h unspecific blocking with 5% anti-donkey serum (Dianova, Hamburg, Germany) with 0.25% bovine serum albumin (BSA; Carl Roth, Karlsruhe, Germany) in PBST at RT on a shaker followed. Subsequently, the primary antibody against GABA derived from guinea pig (Protos Biotech, New York, USA, Cat# NT 108, RRID:AB_2314455) was applied at 1:200 dilution in 5% anti-donkey serum and 0.25% BSA in PBST (pH 7.4) for 2–5 days on a shaker at 4 °C. Alternatively an antibody against GABA, also from guinea pig (ab17413, Abcam, Cambridge, UK) was used at concentrations between 1:1000 and 1:200. Afterwards, 5 times washing for 20 min each in PBS with 1% Triton were followed by application of the secondary antibody, either donkey-anti-guinea pig-Cy3 (1:300 or 1:500; Jackson Immuno research lab, Ely, UK; code: 706-165-148; lot 127715) or donkey-anti-guinea pig-Cy2 (1:300 or 1:500; Jackson Immuno research lab; code: 706-225-148; lot 81590) diluted in PBST for 36 to 60 h on a shaker at 4 °C. Finally the sections were washed 5 times for 10 min in PBST, then 3 times for 5 min in PBS, transferred to slides, covered with Dabco (Sigma Aldrich) and analyzed by means of epifluorescence (Leitz DM RB, Wetzlar Germany) and confocal imaging (Leica SP8 AOBS).

**Fig. 1:**
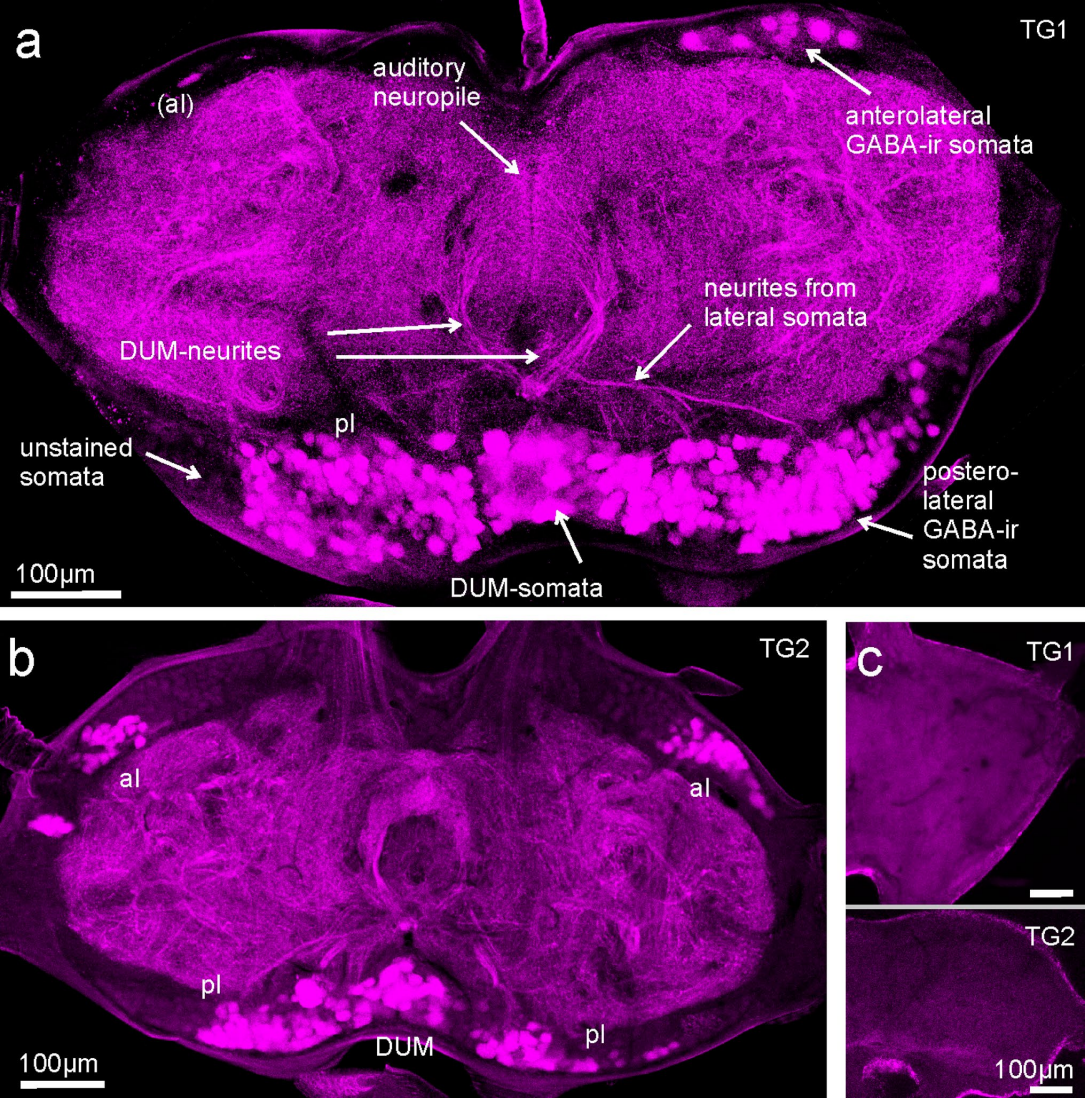
GABA-immunostaining and controls in horizontal sections of prothoracic (TG1) and mesothoracic (TG2) ganglia of *A. nigrovittata*. **a** Typical distribution of immunoreactive somata and neurites in a 280 µm horizontal section showing medially located posterior somata (DUM) neighbored by large groups of posterolateral somata (pl), while anteriolateral somata (al) are only visible on the right side. Neurites of DUM-neurons extend from a posterior branching point towards the auditory neuropile. Other neurites projecting from posterolateral somata towards the midline can be seen as well. *al *anterolateral somata [not included on the left side = (al)], *pl* posterolateral somata, *DUM* dorsal unpaired medium somata. **b** GABA-immunostaining in a 280 µm section of the mesothoracic ganglion (TG2). Stained somata can be located in the typical locations—al, pl, DUM—as in **a**. **c** Control without primary antibody, 160 µm sections of TG1 and TG2

Controls were treated as described above, but without primary antibody. Laser intensity, photodetector sensitivity (Leica Hybrid detector HyD) and pinhole size (1 airy units or 0.6 airy units) varied, but in controls generally were similar to or higher than in test slides. Exemplary values for Fig. 1 are: tests: (a) laser 561 nm 15%, HyD 34%, pin hole 1; (b) laser 561 nm 1.6%, HyD 34.0%, pin hole 1; controls: (c TG1) laser 561 nm 9.8%, HyD 31%, pin hole 0.6; (c TG2) laser 561 nm 13.4%, HyD 69.0%, pin hole 0.6.

In the case of intracellularly stained DUM neurons, stainings were performed as described above, however following the treatment described in “Recordings and staining techniques”. Ganglia were fixed for 1 h on PFA and washed in PBS for 10 min after dissection. Samples were stored at 4 °C for 11–45 days before staining. In case of neurobiotin, streptavidin-Cy2 or streptavidin-Cy3 was added at a dilution 1:200 to the solution containing the secondary antibody.

### Acoustic stimulation

Carrier frequency and temporal pattern of songs play a decisive role in the duetting behaviour of *A. nigrovittata*. For testing frequency tuning a series of block stimuli between 4 and 46 kHz (10 frequency values) were presented in pseudorandom order 5 times each. For tests of varied temporal patterns modelling the male song, two types of programs were presented, either with pause duration varied or with pulse duration varied (data not shown). The stimuli were filled with white noise at 70 dB SPL. A pulse group in the pause program included between 4 and 23 pulses (7 ms duration, ramps 0.5 ms) separated by pauses between 2 and 61 ms (seven values tested) with a total stimulus length of ca. 200 ms in pseudorandom order. Each parameter combination was presented 5 times. The setup and stimulation for measuring frequency responses is described in more detail in Lefebvre et al. (2018). The sound pressure level was calibrated with an accuracy of ± 2 dB with a continuous sound using an amplifier (type 2610, Bruel & Kjær, Nærum, DK) and calibrated Bruel & Kjær condenser microphones (1/2" or 1/4"; orientation directly towards the speaker from the position of the animal, grid on or off according to description).

### Physiological data evaluation

Intracellular data were analyzed using custom-made scripts in Spike 2 (CED). For measuring the amplitude or area of graded potentials intracellular recordings were processed in the following way: (1) The intracellular signal was low-pass filtered with a time constant of 0.5 ms. (2) Action potentials were removed by a high-pass filter (0.2 Hz Butterworth, 1st order). (3) Negative portions of the signal were removed by summing a rectified and not rectified signal. The result was divided by 2 and subtracted from the original signal. (4) Finally, the mean membrane potential during 100 ms preceding a stimulus (“null line”) was calculated and used as reference for defining positive/depolarizing and negative/hyperpolarizing potentials. For calculating peak amplitudes or latencies, graded potentials were only considered if their peak amplitude was higher or lower than the mean membrane potential ± 3 SD. Latencies of graded potentials were only measured if an unequivocal onset of the graded potential starting from below (EPSP) or above (IPSP) the null line was detected. For counting spikes a threshold was defined manually from a high pass filtered (0.2 Hz) signal; the time of a rising or falling amplitude crossing the threshold was used to define spike occurrence. However, whenever equivocal spikes of varying amplitudes (e.g., in bursts or on fast potential changes) were seen, spike numbers were not evaluated.

For assessing the broadness of frequency tuning scans with 10 frequencies between 4 and 46 kHz were used (independently at 50 and 70 dB SPL). First the graded responses were normalized with the peak response at any of the tested frequencies being 100%. Then the kHz values at 50% of the rising and falling flank of the tuning curve were calculated as lower and upper limit. The difference between the two 50% values in kHz defined the broadness of tuning. If the response level at lowest or highest tested frequency (4 and 46 kHz) was above 50%, 4 or 46 kHz were taken as limit (thereby underestimating the broadness of tuning). If the 50% level was crossed more than twice, the two most distant values were taken. For assessing the broadness of temporal tuning, the same logic as for frequency was followed with scans testing seven pulse durations between 2 and 61 ms. The pause duration (ms) values at 50% of the normalized graded responses and the broadness of temporal tuning as difference of the two values were calculated. If the response level at 2 or 61 ms pause duration was above 50%, 2 or 61 ms were taken as lower/upper limit.

For statistical tests Origin Pro (Northampton, MA, USA) was used. For Gaussian distributed data with equal variance *t* tests were applied, for the remaining data Mann–Whitney *U *tests.

Figures were produced using Corel Draw Graphics Suite 2018.

### Pharmacological treatments

After successful penetration of a neuron it was characterized physiologically. Thereafter, three drops of 10^−3^ M picrotoxin (PTX, Sigma Aldrich) dissolved in ringer or water were applied by means of a syringe with a small needle (inner diameter ca. 0.2 mm, leading to rather constant drop size) into the mixture of hemolymph and saline in the prothoracic cavity (for details see Stumpner 1998). For about 1 min the neuron was also hyperpolarized for weak staining of the cell. After 2 min the same physiological tests were performed as before, often several times to see whether the onset of PTX-effect might have been delayed. Subsequently, the neuron was stained for several minutes with hyperpolarizing current. In controls, ringer solution was dropped on the ganglion instead of PTX. Altogether, 40 experiments with application of PTX leading to an estimated concentration of < 10^–4^ mol/l and 29 experiments with ringer application as controls were performed.

## Results

### Immunohistochemistry

Our first aim was to test, whether GABA-immunostaining in a bush-cricket prothoracic ganglion reveals typical auditory DUM neurons and, if so, how many there are. Horizontal vibratome sections of the TG1 showed immunolabelling on all levels when permeabilization was sufficiently long. In sections that contain portions of DUM-neurons (*N* = 34), five areas with GABA-immunopositive somata were stained—two bilateral ones and one medial (Fig. 1a). These included (i) up to ~ 45 anterolateral somata on each side; (ii) up to about 100 posterolateral somata on each side; this area may nearly fuse with (iii) up to about 50 posterior medial somata, which comprises unpaired neurons including DUM-neurons. In dorsal sections or thick sections which included most of the dorsal surface as well, a sixth medially located group (in direct transition to the posteromedial somata) was seen containing 20 or more stained somata. A very similar pattern was found in sections of the mesothoracic ganglion (TG2; *N* = 24; Fig. 1b). Controls without primary antibody (*N* = 12; Fig. 1c), showed stronger unspecific staining only in the ganglionic sheath. In four controls (TG1 and TG2), however, up to ten posterolateral somata expressed several weak dots in the cytoplasm or cell membrane and also in neurites projecting towards the ganglionic center (online resource Fig. ESM1). In at least two GABA-immunostainings these neurons were visible as well. Their staining intensity was lower and staining pattern different (spotty versus more homogeneous) from GABA-positive somata. In conclusion, one typically finds at least five distinct groups of GABA-positive neurons the one in the posterior medial area likely including auditory DUM-neurons.

**Fig. 2:**
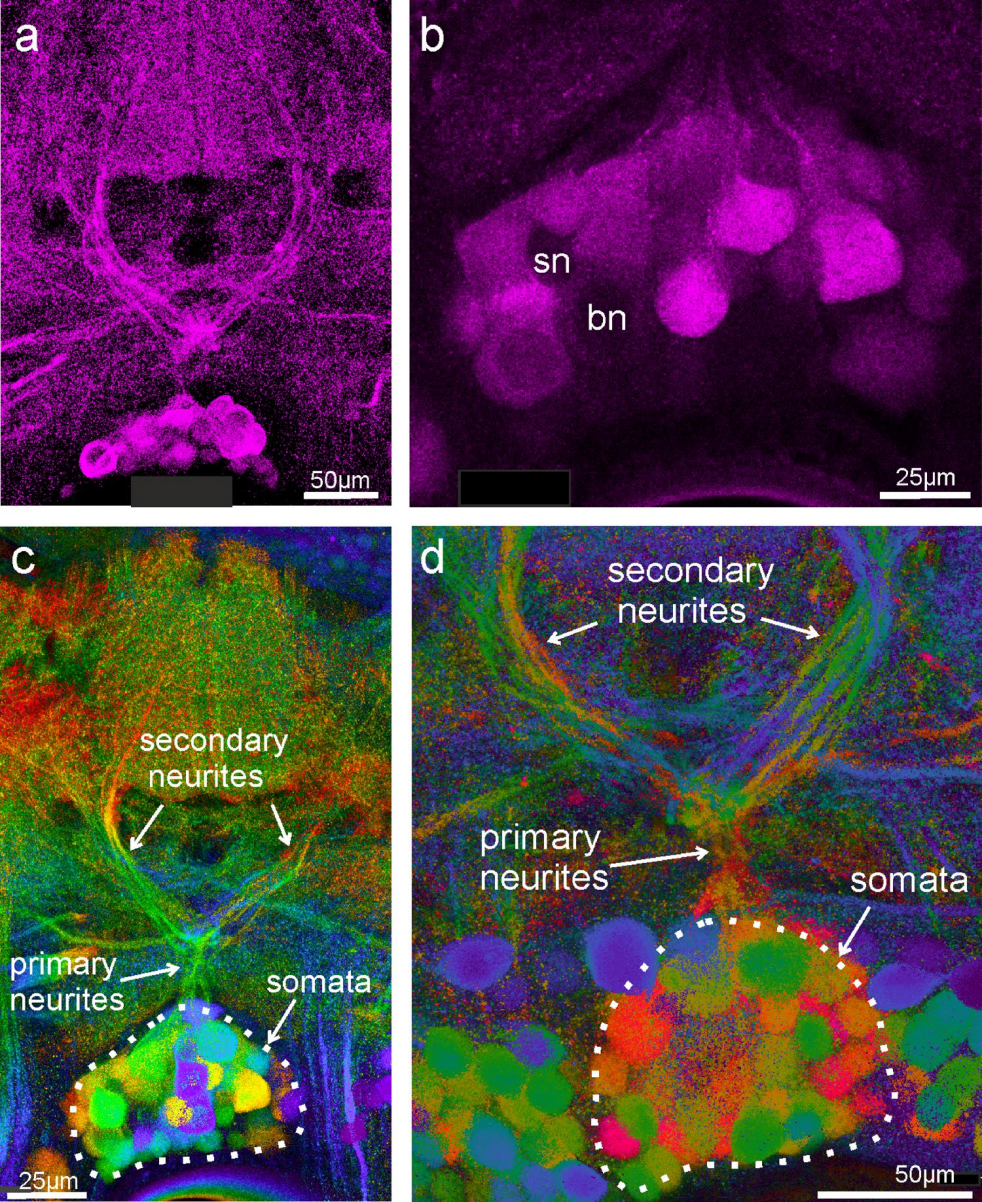
GABA-immunostaining in horizontal sections of TG1 showing structures most likely belonging to auditory DUM-neurons (maximum projections of confocal stacks). **a** View of the central area of TG1 with DUM neurites. At least nine neurites can be separated belonging to nine DUM neurons. **b** Optical section of a soma region indicating the existence of small (sn) and large (bn) GABA-negative somata within the DUM-cluster. **c** and **d** Depth color coded confocal projections (warm colors ventral, but section slightly oblique) showing a well separated cluster of at least 32 medial somata, primary neurites originating from that cluster and at least nine secondary neurites of typical auditory DUM neuron shape projecting towards the auditory neuropile. In **d** up to 47 DUM somata and at least nine secondary neurites exist (same specimen as in Fig. 1a; depth color coding with warm colors for dorsal

**Fig. 3:**
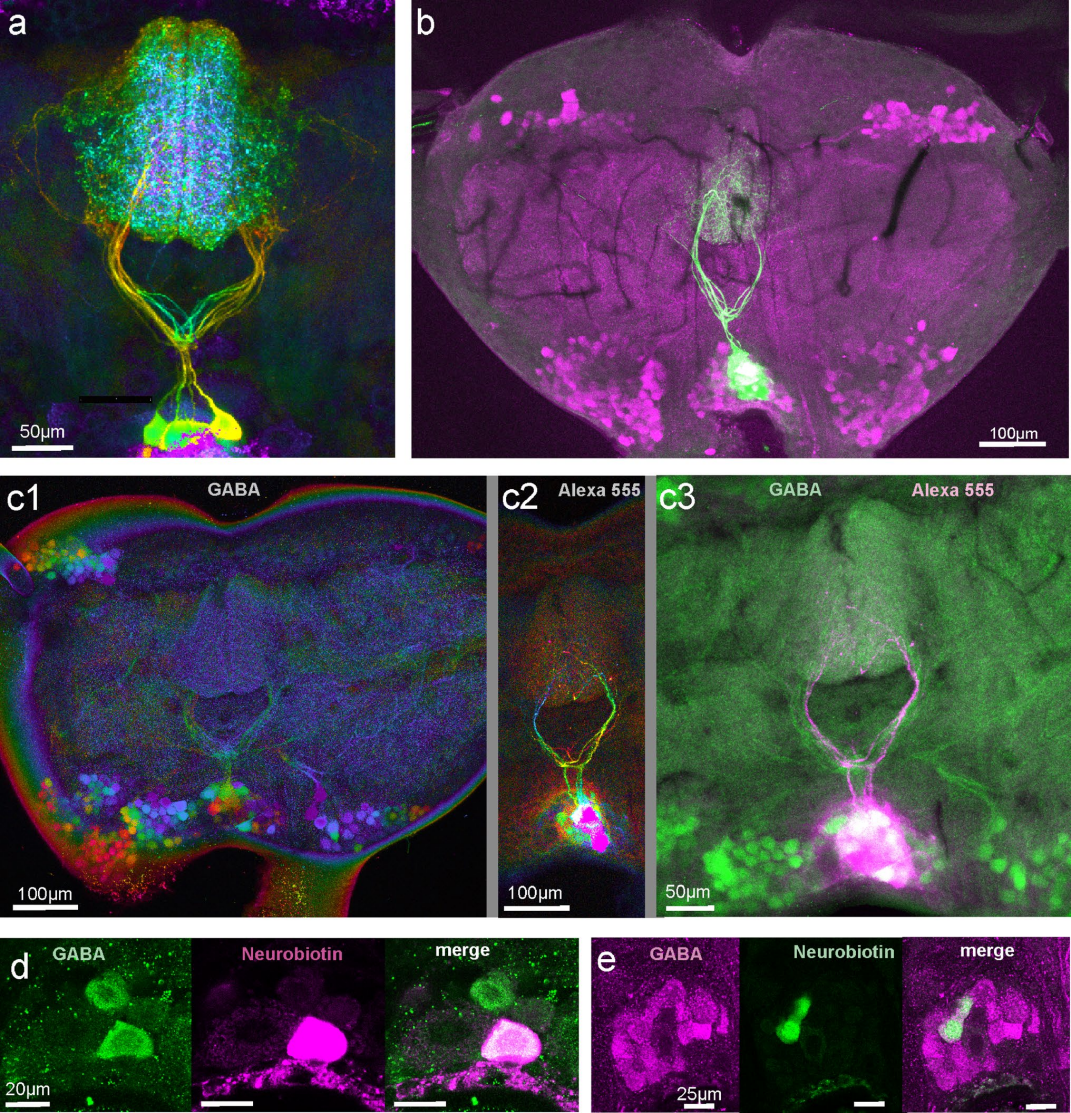
Intracellularly filled DUM neurons combined with GABA-immunostainings. **a** Neurobiotin staining (visualized with streptavidin-Cy2) of somata from at least nine DUM neurons and neurites from four of them in a male *A. nigrovittata*. Depth color coded projection (warm colors: dorsal, for example secondary neurites; more ventral tertiary neurites are green). **b** Lucifer yellow (green) filled DUM neurons (9 somata and at least 3 secondary neurites visible) in a 160 µm horizontal section with GABA immunostaining (magenta) demonstrating the location of DUM somata within the posterior medial cluster of GABA-positive somata. **c** GABA-immunostaining (c1) and Alexa 555 filled DUM neurons (c2) in a 120 µm section. The merge in c3 (GABA green, Alexa magenta) shows the intracellularly filled somata and secondary neurites amidst the GABA-positive structures. **d** One optical section showing a Neurobiotin filled DUM neuron (Cy3, magenta) as one of two GABA positive somata (Cy2, green). One big soma to the left of the Neurobiotin-filled soma is GABA-negative. **e** One optical section showing two Neurobiotin filled somata (green) as GABA-positive among other GABA-positive DUM somata. Two somata next to the Neurobiotin-filled somata are GABA-negative

Determining the exact number of GABA-positive DUM somata in the posterior medial cluster was difficult for the following reasons: (i) the borders between posteromedial and posterolateral groups were hard to determine in many (but not all, see Fig. 3b) sections, confounding a precise counting. (ii) Additionally, often no clear separation to more anterior dorsal median somata existed. (iii) The intensity of staining varied considerably between somata in the same section (see Figs. 2a,b, 3; online resource movie ESM2/M1). However, weaker stained somata with few exceptions were clearly different from background staining of somata in unstained regions.

Between 12 and 50 or more GABA-positive posterior medial somata have been counted in sections of one individual (*N* = 22). In eight of the 22 preparations between 30 and 40 somata were counted. The highest number counted in one section was 47 (see online resource movie ESM3/M2). The highest numbers distributed over two sections were 53 and at least 50. Up to five large unstained somata were seen in the posteromedial cluster (*N* = 13; mean 2.8 ± 1.5 cells; Figs. 2b, 3d; see also movies). In one preparation, two large somata appeared very weakly stained by GABA-antibodies. Also up to five small, unstained somata were present among the medial GABA-positive ones (mean 2.2 ± 1.8 cells; Figs. 2b, 3e). Hence, 35–50 small GABA-immunoreactive DUM cells exist in the posterior medial cluster as well as 2–5 small GABA-negative and 2–5 large GABA-negative somata—the latter likely containing octopamine (Bräunig and Pflüger 2001).

Do GABA-immunostainings reveal neurons with typical morphology of auditory DUM neurons (Lefebvre et al. 2018)? Auditory DUM neurons have a posterior medial soma, a primary neurite projecting anteriorly in the midline, then branching into a secondary U-shaped neurite, which projects in both hemiganglia into the auditory neuropiles (Lefebvre et al. 2018; see also Fig. 3a). Close to the first branching point an additional ventrally orienting branch divides into another but thinner U-shaped tertiary neurite reaching the auditory neuropile on both sides. In horizontal sections that included the central level of TG1 or TG2, typical branches in the shape of local auditory DUM-neurons like primary neurites and secondary neurites were visible in (Figs. 1a, 2, 3c). In sections that included the correct layer and were sufficiently thick, both levels of anteriorly directed secondary and tertiary neurites were visible (see Fig. 3a: orange and green neurites; see also online resource movie ESM3/M2 and Lefebvre et al. 2018). Counting the number of neurites might help assessing the number of DUM neurons. Up to ten primary neurites were discernible as originating from the pool of somata and up to ten secondary and tertiary neurites were visible. The neurites of the different DUM neurons were running close together and were intermingled (Fig. 2a, c, d; see also movies), however, so that counting often was difficult. More laterally running secondary neurites as in type “outside” (Lefebvre et al. 2018) were not among the counted ones, because they are harder to distinguish from crossing branches of bilateral neurons (see Figs. 1a, 3c). Scanning through confocal stacks (for example online resource movie ESM3/M2) indicate, however, that at least five such posteriorly situated secondary DUM neurites might exist. In conclusion, GABA-staining revealed the morphology of typical auditory DUM neurons; a maximum of ten were counted, but additional DUM with more posterior branching exist.

Are all intracellularly marked auditory DUM neurons GABA-positive? To answer this question, GABA-staining was applied on sections from 22 animals with intracellularly stained DUM-neurons. Figure 3a gives an example of several Neurobiotin-filled DUM neurons (depth color-coded) to demonstrate the similarity with DUM neurons showing up in immunostaining as in Figs. 1a, 2a, c. Figure 3b shows nine intracellularly stained DUM somata with at least three stainings also in the neurites. In this specimen, the GABA-immunostained somata show the typical five locations and the somata of the intracellularly recorded neurons are among the group of medial GABA-immunoreactive somata. GABA-staining did not reveal neuropilar structures well in this specimen. Figure 3c shows depth color-coded GABA-staining (c1) and intracellularly filled DUM neurons (Alexa 555; c2) laying well among GABA-immunoreactive somata and neurites (c3). The most consistent results when comparing intracellularly stained somata with GABA-staining in somata come from experiments with neurobiotin as intracellular dye (*N* = 10; 72 cells stained; see Table 1). Fifty seven somata were GABA-positive (some very strong, see Fig. 3d, but many also rather weak), while 15 were GABA-negative (one animal: GABA-staining was not bright, and 6 of 11 neurons were equivocal—counted as negative here). It appeared in some animals, however, that strong intracellular Neurobiotin staining correlated positively with strong GABA-immunostaining. Therefore, control experiments with backfilling large numbers of interneurons from the neck-connective or from a peripheral nerve with Neurobiotin and subsequent GABA-immunohistochemistry were performed. The results did not show GABA-positive Neurobiotin-stained somata in otherwise GABA-negative areas. In fact, in two animals and two different sections, only two of ~ 75 Neurobiotin-stained somata were “GABA-positive”, with about 180 GABA-positive somata not containing Neurobiotin in the proximity. Hence, identified auditory DUM neurons were mostly GABA-positive and this was not an artifact.

**Table 1:** GABA-immunoreactivity of DUM neurons stained intracellularly with Neurobiotin

Experiment no.	No. of cells stained	No. of GABA-positive somata	No of GABA-negative somata
1	1	1	0
2	1	1	0
3	2	1	1
4	5	5	0
5	9	9	0
6	10	10	0
7	11	5	6? very weak immunostaining
8	11	7	4
9	11	9	2
10	11	9	2
Sum	72	57	15

GABA-immunostaining in the mesothoracic ganglion (*N* = 27) revealed DUM morphology as well. Between 6 and 40 somata were seen, while up to seven anteriorly directed primary or secondary neurites were visible. Gross “DUM-morphology” was similar to that of auditory DUM neurons in TG1; the extension of the anterior neuropile was smaller giving the DUM neurites and neuropile a more ring like appearance. Auditory DUM neurons have not been described in the TG2.

### Blocking inhibition

**Fig. 4:**
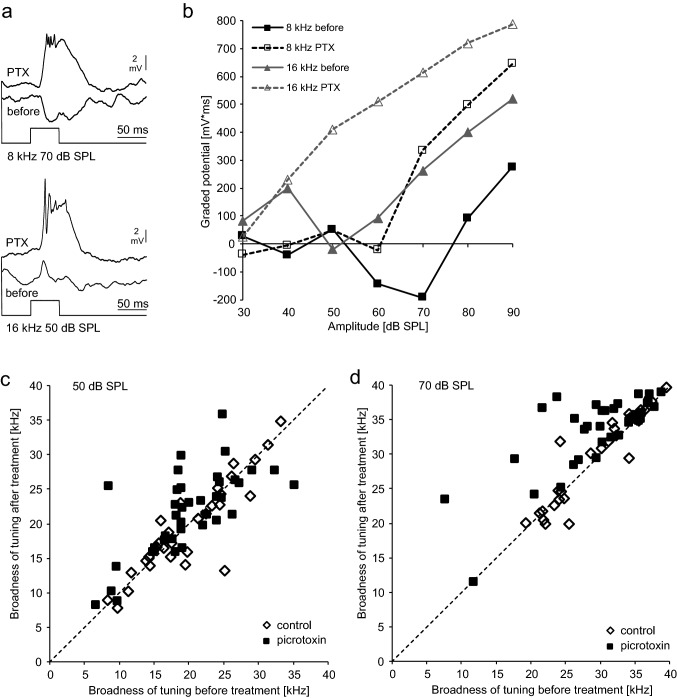
Changes of DUM neuron tuning after application of picrotoxin (PTX). **a** Inhibition at 8 kHz 70 dB SPL and a very weak response at 16 kHz 50 dB SPL are transformed into strong excitation after application of PTX. Averages of five responses, action potentials not clipped. **b** Quantitative data from the cell in **a**. **c** Broadness of tuning (50%-value of normalized responses between 4 and 46 kHz) after treatment with PTX (filled squares) or ringer solution (open diamond) at 50 dB SPL. Data on the dashed line represent no change by treatment. **d** Same neurons as in **c**, but tested at 70 dB SPL

**Fig. 5:**
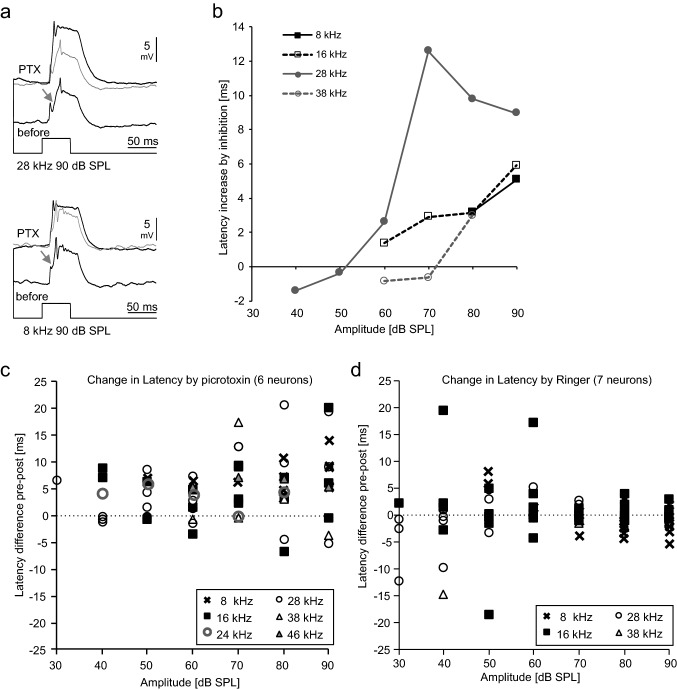
Changes of DUM neuron response onset after application of picrotoxin (PTX). **a** Responses with a clear IPSP in the rising phase at 8 kHz and 28 kHz 90 dB SPL are transformed into responses with smooth rise and corresponding decrease of latency of the first action potential. Averages of five responses, action potentials not clipped. Traces in grey show the response before PTX for easier comparison. **b** Quantitative latency data from the neuron in **a** at various intensities and two more frequencies. **c** Differences of latencies of the first action potential before (“pre”) minus after application of PTX (“post”). Therefore, a positive value means that the latency was longer before treatment. Data of six neurons at the given intensities and up to four different frequencies. Values were calculated only at those intensities and frequencies, at which an unequivocal action potential occurred at one to five stimulus repetitions (values are the differences of the means). The broken line represents 0 ms-change. **d** As in **c**, but data from seven neurons with application of ringer solution

**Fig. 6:**
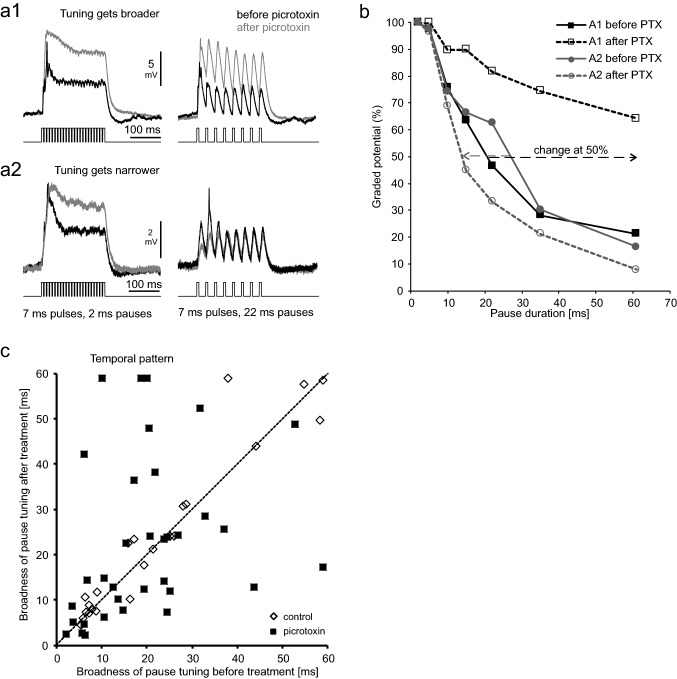
Effects of picrotoxin and controls on temporal processing (pauses varied, pulses constant at 7 ms). **a** Shows response traces of two neurons (a1, a2) before (black) and after application of PTX (gray) at two different stimulus patterns. **b** Graded potentials of the two neurons shown in **a** at various pause durations. The broken arrows indicate a broadening (black) or narrowing (gray) of temporal tuning. **c** Broadness of tuning (50%-value of normalized curves) before and after treatment with PTX (black squares, 35 neurons) or ringer-solution (open diamonds, 23 neurons). The broken line represents no change in broadness

Which impact does inhibition have on auditory DUM neuron responses? Responses of DUM neurons show clear signs of inhibition like inhibitory potentials or reduced activity at high intensities (Lefebvre et al. 2018). The inhibition of sound-evoked responses of other auditory neurons of *A. nigrovittata* is at least partly picrotoxin (PTX)-sensitive (Stumpner 1998; Molina and Stumpner 2005). We thus tested, whether DUM neurons are also sensitive to PTX-applications (Figs. 4, 5, 6). Sound-evoked inhibitory postsynaptic potentials (IPSPs) as well as negative deflections in the rising flank of the graded potentials have been eliminated or strongly reduced in 10 out of 14 DUM-neurons after application of PTX, in contrast to application of ringer (Figs. 4a, 5a). PTX-application uncovered excitation at frequencies and intensities that elicited IPSPs before the treatment, thus shifting the response thresholds to nearly 20 dB lower values (Fig. 4b). The width of frequency tuning following PTX application was on average broader and significantly different from controls at 50 and 70 dB SPL. As evident from the large variation, not all cells changed their tuning in response to PTX treatment. For example, only 18 of 39 cells broadened their tuning by more than 2 kHz at 70 dB SPL (mean 7.9 ± 4.7 kHz). At 50 dB SPL, the average tuning broadened following PTX application by 1.99 ± 4.76 kHz (*N* = 40) and following ringer application by only 0.40 ± 3.41. kHz (*N* = 29; *U* test, *p* = 0.022). At 70 dB SPL, the average tuning broadened with PTX by 3.92 ± 4.88 kHz (*N* = 39) and with ringer by 0.17 ± 2.30 kHz (*N* = 27; *U* test, *p* < 0.001).

Negative deflections occurring in the rising flank, interpreted as IPSPs before (Lefebvre et al. 2018; Stumpner et al. 2019), were abolished by PTX -application (Fig. 5a). Consequently, latencies of first spikes (when the neurons produced action potentials at all) were reduced at most intensities (Fig. 5b–d). The first-spike latencies (Fig. 5c, d), averaged for each neuron from all frequencies and intensities, decreased by 4.43 ± 3.44 ms after PTX-application and increased by 0.44 ± 1.01 ms after ringer (*N* = 6 for PTX, *N* = 7 for controls; significant difference, Mann–Whitney *U *test, *p* = 0.018). With PTX, largest differences occurred at higher intensities, where inhibition was strongest (Fig. 5c). With ringer, largest differences occurred at lower intensities (where responses were more variable; Fig. 5d).

Blocking inhibition also affected temporal processing in many DUM neurons. When stimulated acoustically with trains of sound pulses having inter-pulse pauses of varying duration (Fig. 6), responses in many neurons got overall stronger, but the strength of the effect depended on temporal pattern (Fig. 6a). The broadness of tuning changed in either way: some neurons broadened their response range of pause durations (Fig. 6a1, black curves in Fig. 6b), in other neurons tuning got narrower (Fig. 6a2, grey curves in Fig. 6b). For the whole data set, there was an enormous spread of response changes following PTX-application, which was not seen with application of ringer (Fig. 6c). When comparing the changes (absolute values of broadening or narrowing) between treatment and control the average change was 12.8 ± 13.8 ms with PTX (*N* = 35) and 3.3 ± 4.5 ms in controls (*N* = 23). This difference is significant (Mann–Whitney *U *test, *p* < 0.001).

## Discussion

### GABA-Immunostaining

DUM-neurons of insects have been described to be either GABA-ergic or octopaminergic (Bräunig und Pflüger 2001). Preliminary data from large series of 16 µm sections (Lefebvre et al. 2018) indicated that there are approximately 50 small GABA-immunopositive somata in the posterior medial prothoracic ganglion, where DUM-somata are situated. Therefore, two types of experiments for testing whether auditory DUM-neurons in *A. nigrovittata* produce GABA have been executed.

First, immunohistochemical experiments were conducted with primary antibodies that have been used in other Orthoptera (Protos-antibody in grasshoppers; Kunst et al. 2011) and in several other invertebrates (Abcam-antibody, *Caenorhabditis elegans*: Gendrel et al. 2016; *Apis melifera*, Held et al. 2016; *Drosophila melanogaster*, Fei et al. 2010). Originally, wholemount staining of complete ganglia has not been successful in *A. nigrovittata* due to penetration problems. The general distribution of GABA-immunoreactive somata is similar to the ones described for locusts (Watson 1986) and crickets (Spörhase-Eichmann et al. 1989). For the cricket prothoracic ganglion, about 350–400 GABA-immunoreactive somata were counted (Spörhase-Eichmann et al. 1989). In *A. nigrovittata* the sum of the anterior, posterior and dorsal groups indicate a similar number.

GABA-positive neurites displayed typical auditory DUM-morphology. A posterior medial group of GABA-positive somata gave rise to unpaired median primary neurites. These neurites divided into the two typical U-shaped secondary and tertiary neurites projecting anteriorly to the edges of the auditory neuropile. This represents fully the described DUM neuron types “narrow” and “loops”, which constitute the majority of auditory DUM neurons (Lefebvre et al. 2018). The stainings of neuropilar structures often were more or less spotty and did not allow precise counting of such neurites, but clearly whole groups of such neurites were visible in the majority of ganglia—in general one could estimate about 5 to at least 10 of such secondary neurites in a prothoracic ganglion section. Additionally about five more posterior DUM neurites potentially belonging to type “outside” DUM neurons (Lefebvre et al. 2018) were visible. The number of GABA-positive somata in one section was larger and varied between about 20 and more than 50. However, several factors hampered exact counting of somata. Somata were mostly distributed over two or more sections. Borders of individual somata, especially when staining was strong, were not always clear. The delimitation of medial posterior somata from the adjacent lateral or dorsal groups was often equivocal. Therefore, it seems safe to state that 35–50 GABA-positive somata of DUM neurons exist, whereby only 10–15 typical neurites of auditory DUM neurons could be resolved. At least a part of the remaining GABA-positive DUM neurons might be intersegmental interneurons responding to other mechanosensory stimuli as in locusts (Thompson and Siegler 1991). Larger (or even smaller) numbers of somata may be due to counting problems, variance of immunostaining, or interindividual variation. The number of GABA-positive posterior medial somata in TG2 was slightly lower (18–40 somata, up to seven neurites). This is not surprising, since the TG2 is not the target of auditory sensory cells. These DUM neurons likely are serially homologues of auditory DUM neurons in TG1 and might have non-auditory mechanosensory function. Studying their responses might foster ideas about the evolutionary starting point and specializations of prothoracic auditory DUM neurons.

Why is the number of visible GABA-positive DUM neurites so much smaller than the number of posterior medial GABA-positive somata? Lefebvre et al. (2018) concluded from identified morphology and physiology that at least 15 auditory DUM neurons exist. This fits quite well to the about 10 neurites representing “narrow” and “loops” and the about five more posterior neurites representing “outside” and “descending” (Lefebvre et al. 2018). Lefebvre et al. (2018) speculated that the group “outside” might contain members with increasingly more posterior secondary neurites and correspondingly more vibrational and less auditory responses. Therefore, a portion of the GABA-positive DUM-somata might belong to such neurons responding mostly to vibration. For the Omega neuron 1 in crickets inhibition by vibration has been described (Wiese 1981). If this inhibition exists also in the Omega neuron of bush-crickets it might arise from DUM neurons. Other GABA-positive DUM neurons might belong to the rarely recorded ascending neurons (Lefebvre et al. 2018). These neurons with a much longer and more dorsal primary neurite would not be classified as typical auditory DUM neuron morphology but still would show up among the somata—but direct evidence for the existence of such cluster is not apparent in the GABA-stainings.

To test, whether identified auditory DUM neurons are GABA-positive, neurons have been electrophysiologically characterized, stained intracellularly and then subjected to immunohistochemistry. Such experiments have been successfully performed before in locust auditory neurons (TN1, Sokoliuk et al. 1989). In Ancistrura, the intracellular dye appeared to have some influence on the quality of immunostaining. The results were most consistent with Neurobiotin with up to 10 of 10 intracellularly stained auditory DUM neurons in one individual ganglion being GABA-positive. In five of ten individuals one or more stained auditory DUM neurons appeared to be GABA-negative. One potential problem was penetration of the antibodies into the slice after longer lasting physiological experiments. GABA-negative DUM somata, however, were also seen without preceding physiological experiments: on average, two to three small somata and three large somata in the posterior median cluster were GABA-negative. The large ones most likely were octopaminergic neurons (Bräunig and Pflüger 2001; Hörner 1999). Watson (1986) has also seen “a few” smaller GABA-negative somata in the posterior group in locust thoracic ganglia. As a conclusion, few small GABA-negative somata may exist in *A. nigrovittata*, and they might belong to auditory DUM neurons. Therefore, it seems safe to conclude that the vast majority of auditory DUM-neurons contain GABA as a transmitter, which fits to their presumed inhibitory nature (Lefebvre et al. 2018). One DUM neuron (“AV4”) in the brain of *D. melanogaster*, which has been described as a member of a set of auditory temporal filters is GABA-ergic as well (Clemens et al. 2015).

### Physiological experiments

DUM-neurons are not only a potential source of inhibition in the prothoracic network, but also the target of inhibitory influences (Lefebvre et al. 2018; Stumpner et al. 2019). Such inhibitions were also described for DUM neurons in grasshoppers (Stumpner and Ronacher 1991) and cave crickets (Stritih and Stumpner 2009). Companion DUM-neurons may cause this inhibition. Picrotoxin eliminated or strongly reduced inhibition in auditory neurons of various bush-crickets (Stumpner 1998, 2002) and did so in DUM neurons as well. Obvious hyperpolarizing response of DUM neurons, which is typical for certain DUM neurons at specific frequencies and intensities (see Lefebvre et al. 2018; Fig. 4) was lost or even replaced by excitation, similar as reported also for the AN1-neuron of *A. nigrovittata* (Stumpner 1998). Concurrently, thresholds decreased leading to an overall broadening of frequency tuning in about 50% of DUM neurons. Therefore, if DUM neurons are the source of inhibition in other auditory neurons, this inhibitory influence is shaped by inhibition as well—at least in some of the DUM neurons. Given the general importance of inhibition (e.g., Pollak et al. 2011) and also the potential interaction between inhibitory neurons in CNS (e.g., Pinaud et al. 2008; Isaacson and Scanziani 2011), such inhibition of inhibitory neurons may be expected, but is rarely directly shown (except for reciprocal inhibition as in the highly directional ON1; Selverston et al. 1985).

A negative deflection in the rising flank of many DUM neuron responses has been interpreted as sign of inhibition (Lefebvrer et al. 2018). Current experiments with picrotoxin corroborated this hypothesis. The negative deflection was lost with PTX and consequently the first action potential’s latency decreased (if action potentials were produced at all). The difference was large in several neurons: at some higher intensities and different frequencies latencies decreased by 10 ms or more. A reduction of latency by blocking inhibition is also known from other systems as the vertebrate retina, where it affects temporal tuning to optical stimuli (Venkatamarani et al. 2014). In *A. nigrovittata*, the function of this IPSP in the flank of the graded potential remains speculative. A delayed action potential or peak of graded potential would—if inhibitory—allow for a longer phasic onset response in the affected neuron and thereby potentially tune the duration of a phasic response to certain temporal features. In crickets, similar arguments have been made for a pair of neurons with mutual inhibition stressing its phasic response (Hedwig 2016; Zhang and Hedwig 2019). PTX-application also changed the temporal processing of pulse repetition rates in many DUM neurons. Interestingly, some became more narrowly tuned to a set of pulse rates, others broader. Such divergent response changes after blocking inhibition are hard to find in the literature (few examples in electric fish, where low pass and high pass neurons showed a range of divergent changes upon PTX-application; George et al. 2011). In conclusion, also a inhibition influencing the temporal pattern of auditory responses of other auditory neurons like AN2 (Stumpner and Molina 2006) is shaped itself by inhibition.

Unfortunately, it was not possible to demonstrate directly that DUM neurons have an inhibitory effect in the network. Few experiments were performed with killing a DUM neuron followed by recording from other neurons, but these were inconclusive. The probability, to find a directly postsynaptic neuron to the killed DUM neuron likely is low while the variability induced by the killing procedure (laser illumination) is rather high. Therefore, a proof for the suggested role of DUM neurons in the network remains to be given.

### Conclusion

A population of auditory DUM neurons exists in the bush-cricket *A. nigrovittata* and constitutes a filter bank for carrier frequency and temporal pattern. Most if not all of these DUM neurons are GABA-immunopositive and therefore a potential source for inhibition of other auditory neurons like AN1, AN2 and AN5-AG7. Responses of many DUM neurons additionally are shaped by inhibition. This stresses the relevance of inhibitory processes in invertebrates—the majority of described auditory neurons in the prothoracic ganglion of *A. nigrovittata* are inhibitory.

## Electronic supplementary material

Below is the link to the electronic supplementary material.

Supplementary file1 (PDF 253 kb)

## Abbreviations

BSA: Bovine serum albumin
DUM: Dorsal unpaired median
EPSP: Excitatory postsynaptic potential
GABA: γ-Aminobutyric acid
HyD: Hybrid detector
IPSP: Inhibitory postsynaptic potential
PB: Phosphate buffer
PBS: Phosphate buffered saline
PBST: Phosphate buffered saline with triton
PFA: *Para*-Formaldehyde
PTX: Picrotoxin
RT: Room temperature
