# Supplementary material for: Auditory DUM neurons in a bush-cricket: inhibited inhibitors
Source: J Comp Physiol A Neuroethol Sens Neural Behav Physiol. 2020 Jul 12;206(5):793–807. doi: 10.1007/s00359-020-01438-2 (PMC7392950; doi:10.1007/s00359-020-01438-2)
Supplement: Supplementary file 1 — Supplementary file1 (PDF 253 kb) [file 359_2020_1438_MOESM1_ESM.pdf]

Andreas Stumpner*, Silvia Gubert, Debbra Y. Knorr, Martin C. Göpfert

# Auditory DUM neurons in a bush-cricket: inhibited inhibitors

University of Göttingen, Johann-Friedrich-Blumenbach-Institute of Zoology and Anthropology, Dept. Cellular Neurobiology; Julia-Lermontowa-Weg 3; D-37077 Göttingen, Germany

*corresponding author: astumpn@gwdg.de

Supplementary material

Figure ESM1: Neurons stained in some controls

ESM2: Stumpneretal-DUM-ESM2-M1.wmv

ESM3: Stumpneretal-DUM-ESM3-M2.wmv

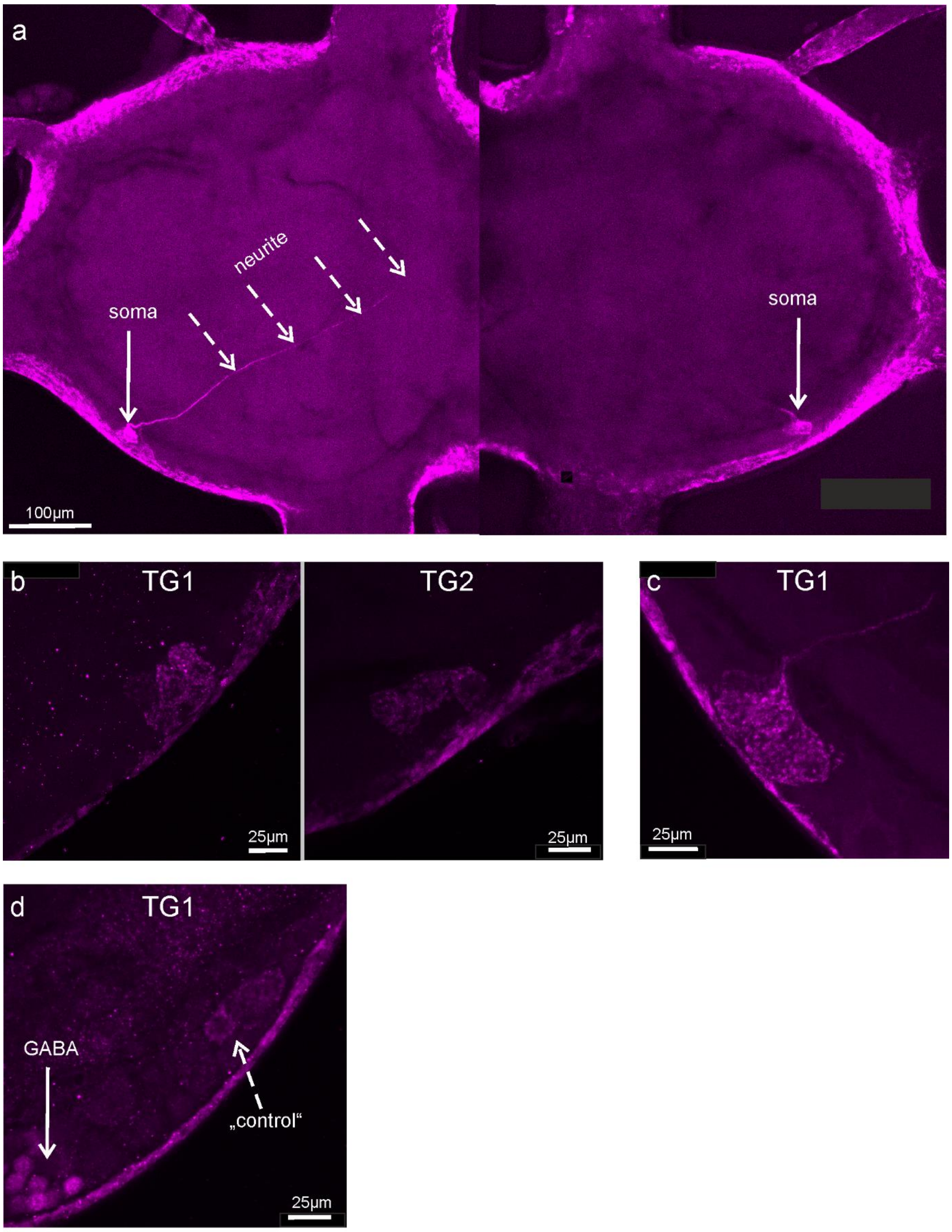


Fig. ESM1

Fig. ESM1: Staining of a group of neurons by secondary antibodies in some control experiments. In the mesothoracic ganglion (A, B right) or prothoracic ganglion (B left, C) a group of up to ca. 10 posterolateral neurons was stained in characteristic spotty fashion. In some preparations, the primary neurites extending towards the center of the ganglion were visible as well. A) is from one ganglion, but the two halves are composed from different z-stack frames, since the section was slightly oblique. D) shows the staining in comparison to GABA-staining, which was brighter and not spotty. Sections are between 70 and 280 µm thick. Settings for laser intensity (relatively high to visualize the neurons), HyD-detector sensitivity and pinhole size (airy units): A) laser 561 nm 13.4%, HyD 37.8%, pin hole 0.6;  B left) laser 561 nm 22.2%, HyD 34.0%, pin hole 0.6;  B right) laser 561 nm 15.3%, HyD 31.3%, pin hole 0.6;  C) laser 561 nm 33.3%, HyD 34.0%, pin hole 1;  D) laser 561 nm 21.9%, HyD 34.0%, pin hole 0.6.

ESM2: Stumpneretal-DUM-ESM2-M1.wmv

Confocal stack of GABA-immunostaining of a 70 µm horizontal section of the prothoracic ganglion of a male *A. nigrovittata*. About 35 GABA-positive medial somata and 8 to 10 primary neurites can be seen of varying staining intensity. At least 5 smaller somata and one (at the end) large soma are GABA-negative.

ESM3: Stumpneretal-DUM-ESM3-M2.wmv

Confocal stack of GABA-immunostaining of a 280 µm horizontal section of the prothoracic ganglion of a male *A. nigrovittata*. The movie starts ventral. First, the tertiary thinner neurites can be seen, followed after crossing of neurites from lateral GABA-positive fibres by the more dorsal secondary neurites and also primary neurites. Two large ventral median somata are likely from octopaminergic DUM neurons (first sections of the movie). About 47 somata and at least 9 secondary or tertiary neurites of DUM neurons can be seen.
